# Prediction of gastrointestinal cancers in the ONCONUT cohort study: comparison between logistic regression and artificial neural network

**DOI:** 10.3389/fonc.2023.1110999

**Published:** 2023-04-24

**Authors:** Rossella Donghia, Vito Guerra, Giovanni Misciagna, Carmine Loiacono, Antonio Brunetti, Vitoantonio Bevilacqua

**Affiliations:** ^1^Data Science, National Institute of Gastroenterology - IRCCS “Saverio de Bellis”, Castellana Grotte (BA), Italy; ^2^Scientific and Ethical Committee Polyclinic Hospital, University of Bari, Bari, Italy; ^3^Department of Electrical and Information Engineering, Polytechnic University of Bari, Bari, Italy

**Keywords:** logistic regression, artificial neural network, machine learning, gastrointestinal cancer, nutrition

## Abstract

**Background:**

Artificial neural networks (ANNs) and logistic regression (LR) are the models of chosen in many medical data classification tasks. Several published articles were based on summarizing the differences and similarities of these models from a technical point of view and critically assessing the quality of the models. The aim of this study was to compare ANN and LR the statistical techniques to predict gastrointestinal cancer in an elderly cohort in Southern Italy (ONCONUT study).

**Method:**

In 1992, ONCONUT was started with the aim of evaluating the relationship between diet and cancer development in a Southern Italian elderly population. Patients with gastrointestinal cancer (ICD-10 from 150.0 to 159.9) were included in the study (*n* = 3,545).

**Results:**

This cohort was used to train and test the ANN and LR. LR was evaluated separately for macro- and micronutrients, and the accuracy was evaluated based on true positives and true negatives *versus* the total (97.15%). Then, ANN was trained and the accuracy was evaluated (96.61% for macronutrients and 97.06% for micronutrients). To further investigate the classification capabilities of ANN, k-fold cross-validation and genetic algorithm (GA) were used after balancing the dataset among classes.

**Conclusions:**

Both LR and ANN had high accuracy and similar performance. Both models had the potential to be used as decision clinical support integrated into clinical practice, because in many circumstances, the use of a simple LR model was likely to be adequate for real-world needs, but in others in which there were large amounts of data, the application of advanced analytic tools such as ANNs could be indicated, and the GA optimizer needed to optimize the accuracy of ANN.

## 1 Introduction

Gastrointestinal (GI) cancer is a term for the group of cancers that affect the digestive system and involve a range of body parts such as the esophagus, stomach, colon, and rectum (1). The WHO stated that approximately 3.5 million new cases of gastrointestinal cancer were diagnosed in 2018 (2). Many studies have shown that an improper diet is associated with an increased likelihood of developing this type of malignancy due to fostering the inflammatory process and the likelihood of microbiome dysregulation (3). Colorectal cancer (CRC) and pancreatic disease is the most commonly diagnosed, with a lower probability of survival than other gastrointestinal cancers, followed by stomach, liver, and esophageal cancer (4, 5). Obesity is a major risk factor, with excessive fat consumption and a paucity of fiber, vitamin, and mineral intake. It has been estimated that by 2025, obesity rates will reach approximately 18% in men and 21% in women (6). The rising rates for this condition will lead to a higher prevalence of gastrointestinal malignancies in the coming years (7). The main cause of obesity is a diet rich in fats and with a low intake of fiber, vitamins, and minerals, in short, an unsatisfactory intake of macro- and micronutrients from food. In the literature, the association between nutrients and different types of cancer has been much discussed, but the molecular mechanisms are still unclear. In recent years, owing to economic advances and a prolonged life expectancy, obesity has become a global health problem, leading to an increase in the prevalence of gastrointestinal diseases that have become chronic (8).

An accurate prediction of clinical outcomes is the basis of successful decision-making and can lead to better patient care. Although clinical prediction might prove valuable, it is challenging for clinicians who must balance the relative contributions of numerous risk factors. Clinicians predict the outcome of a disease or adverse event by using probabilities with heuristic methods on the basis of training and experience. Although these heuristic methods may be necessary and useful, they can be biased and lead to systematic errors. To decrease systematic errors and allow the improvement of care, the use of artificial intelligence has been widely used, and several articles analyze the differences among statistical approaches applied to different pathologies (9–11).

Machine learning, a subgroup of artificial intelligence, is widely used in clinical medicine for cancer detection, diagnosis, and classification (12). In fact, since, the 1960s, machine learning algorithms were used to analyze and interpret cancer (13).

The aim of this study was to compare empirically and describe the predictive ability machine learning methods, i.e., of Logistic Regression (LR) and Artificial Neural Network (ANN) in the predicting GI cancer in epidemiological research in a previously unstudied historical cohort in order to assess the discrimination capabilities of such two techniques, now widely used in clinical settings.

**Figure 1:**
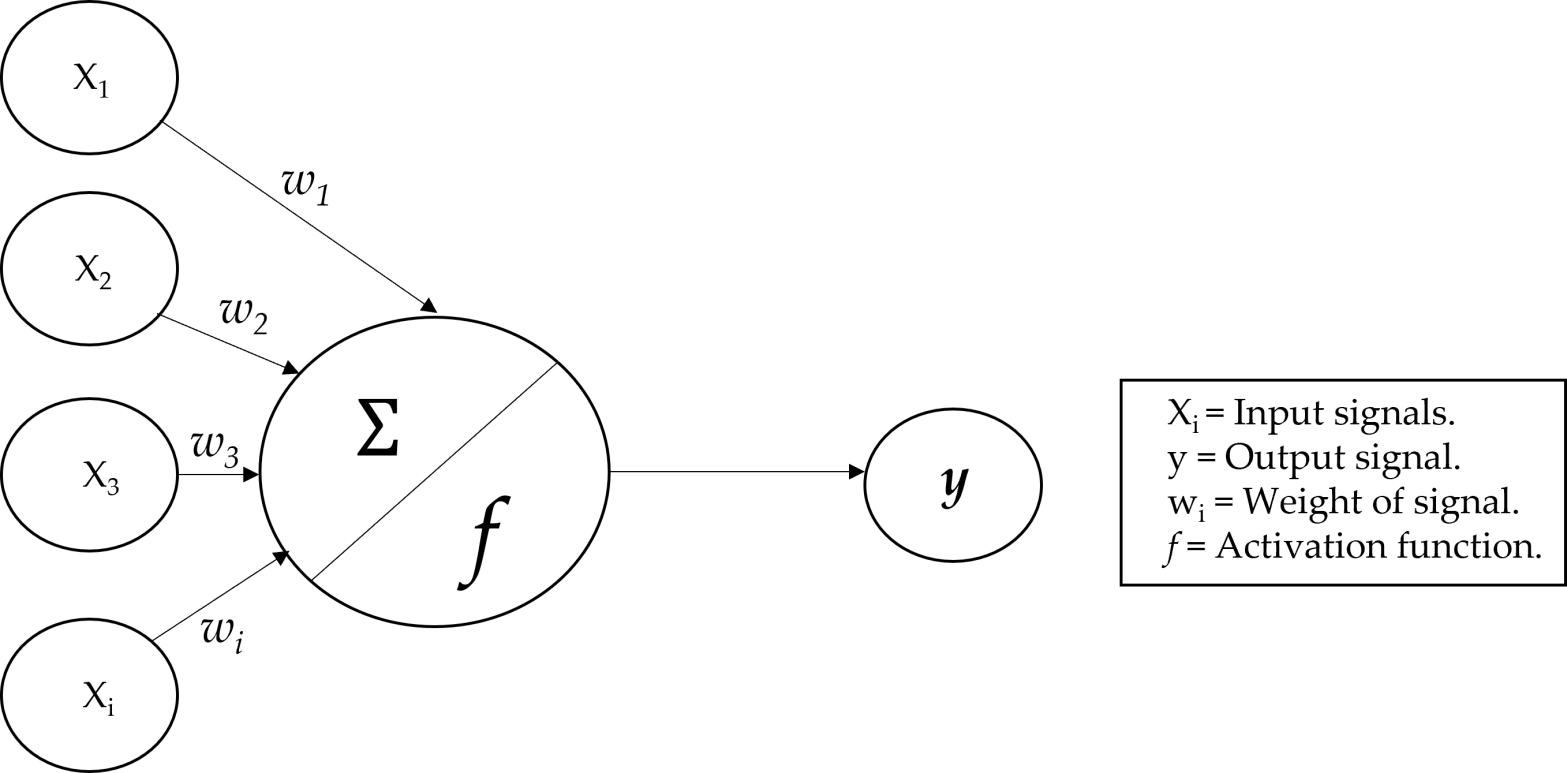
The ANN architecture.

An analysis of nutrient variables (micro- and macronutrients) was performed in the first step with LR, calculating the accuracy, and then with ANN to compare which statistical method could predict gastrointestinal cancer more accurately. The power of ANN was to recognize the relationships between covariates and response variables *via* a learning process (14), as compared with the classical statistical method used. There is evidence that ANN is a better predictive model than the classical linear and logistic models in several clinical fields (15) and that it is superior to classical linear methods for the identification of a clinical outcome in patients (16). ANNs, emulates human neurons; their connections, are built on the nodes that receive input data, process them, and are able to send information to other neurons (17). Dendrites receive signals from other neurons, and the neuron cell body keeps all the input signals to generate output (18). The model of neurons in ANNs can be explained in **Figure 1**, where it is shown the perceptron model.

There are various types of ANN architecture (19), but we used the multilayer perceptron, which is a more complex implementation, based on the perceptron model, which demonstrated to be more efficient than traditional statistical techniques (20). The output of the model is a signal based on the function of the sum of inputs. In this case, the output was the probability of the input being a predictor of gastrointestinal cancer.

The ANN is a directed network defined as the relationship between the input signals coming from the dendrites (*x_i_* variables) and the output signal (*y* variable). As in the neuronal biological system, each dendrite has a weight (*w_i_*) that represents the importance with respect to outcome. Moreover, *f* is the activation function, based on the sum of the input signals (21).

Logistic regression is a statistical method applied to evaluate the relationship between potential risk factors and clinical outcomes and to control the effect of variables associated with risk factors and clinical outcomes (22, 23). As for linear regression, *α* and *β* are the estimates of coefficients (24).

A confusion matrix is a more common way to describe the results of studies based on classifiers. A confusion matrix is a *k* × *k* contingency table, and a binary confusion matrix is a special case when there are only two classes: C (positive class) and not-C (negative class).

In a binary confusion matrix, observations classified correctly into the positive class are called true positives (TPs), and observations classified correctly into the negative class are called true negatives (TNs). Instances of the positive class misclassified as negative are called false negatives (FNs) and instances of negative the class misclassified as positive are called false positives (FPs) (25). From these frequencies, one can calculate classification performance indicators that reflect how the classifier performs in detecting the given class. The most common of such indicators are as follows:

## 2 Materials and methods

### 2.1 Participants

In 1992, the ONCONUT prospective cohort was started with the aim of evaluating the relationship between diet and cancer development in a Southern Italian elderly population (*n* = 35,000). The study was sponsored by the Italian National Institute of Health and carried out by the Epidemiology and Biostatistics Laboratory of the National Institute for Research in Gastroenterology “Saverio De Bellis” (26). The internal ethics committee of “S. de Bellis” Hospital agreed with this study. This study was designed in accordance with the general ethical principles outlined in the Declaration of Helsinki. The number of the ethics committee and its informed consent were not available as. This is a retroactive historical cohort. Over 30 years ago, the ethics committee was internal to the institute; therefore, the acceptance of the project was without intermediaries and the number did not exist. The scientific director directly gave consent for the use of the archived data and their future publication. Furthermore, this type of study, being a description of a historical cohort with statistical applications, did not require an ethics committee, but a description of the situation is necessary as described above (27).

From 1 April 1992 to 31 July 1993, patients referred to the Clinical Pathology Laboratory of the three USL BA 16 areas (Municipalities of Monopoli and Polignano a Mare), BA 17 (Municipalities of Gioia del Colle and Santeramo in Colle), and BA 18 (Municipalities of Castellana Grotte, Turi, Putignano, Noci, Alberobello, and Locorotondo) were estimated to be 11,622, but only 5,632 (48.46%) (ONCONUT 1) completed approximately 90% of the semiquantitative food frequency questionnaire (FFQ). After 5 years, 4,563 patients returned (ONCONUT 2). After excluding cases other than those of gastrointestinal disease (other types of cancers), 3,545 (77.69%) presented complete data for analysis.

The prevalence of gastrointestinal cancer (ICD-10, codes from 150.0 to 159.9) during the years 1992-1993 (ONCONUT 1) was considered the main outcome. Only 2.85% of them developed gastrointestinal disease. Food conversion into nutrients (macro- and micronutrients) and calories was performed using the Italian National Institute of Nutrition Food Composition.

Tables were integrated with data from Fidanza (28), using a validated semiquantitative FFQ administered to the participants. The glycemic index (GI) derived from each food (29) was calculated using tables and the glycemic load (GL), as suggested in the study of Foster-Powell et al. (30).

All participants signed informed consent before the examination, and general approval of the studies was obtained from the IRB of the head institution, the National Institute of Gastroenterology and Research Hospital “S. de Bellis” in Castellana Grotte, Italy. The studies were conducted following the 1975 Helsinki Declaration. The present investigation was conducted following the “Standards for Reporting Diagnostic Accuracy Studies” (STARD) guidelines, and the manuscript was organized following the “Strengthening the Reporting of Observational Studies in Epidemiology - Nutritional Epidemiology” (STROBE-nut) guidelines (31).

### 2.2 Statistical analysis

Patients’ characteristics are reported as mean ± standard deviation (*M* ± SD) for continuous variables and as frequencies and percentages (%) for categorical variables. For testing the associations between groups, the chi-square test or Fisher’s exact test for categorical variables was used, as necessary, while the Wilcoxon rank-sum (Mann–Whitney) test was used for continuous variables. The proportions test was used to evaluate differences in accuracy between the two compared methods.

Gastrointestinal cancer was used in the models as a dependent variable, and macro- and micronutrients were used as independent variables, while gender and age, the most important epidemiological variables, were used to correct both models.

We split randomly the data into the training and testing subgroups for ANN. The training data included 75% of the samples (*n* = 2,659), while the remaining data, the test data, accounting for 25% (*n* = 886) were used to test the model.

#### 2.2.1 Logistic regression

It was included in the family of generalized linear models (GLMs). It was a statistical technique conducted to find the most relevant model when aiming to study the relationship between an outcome (dependent or response variable) and a set of independent variables (predictors or explanatory). What distinguishes the logistic from the linear model was the nature of the dependent variable, which can be of a binary (or dichotomous) type and, as such, assume the values of 0 or 1. Logistic regression defined whether the dependent variable belongs to one group or another. The values that were assigned to the levels are based on the probability that a given subject belongs to less than one of the two groups, therefore only in a range of values included in the interval (0,1). The dependent variable was a variable with a Bernullian random distribution. This model was tested on the total cohort because it is very easy to carry out and achieves a very good performance.

All variables separated into macro- and micronutrients with age and gender as covariates were included together in the model. After logistic regression, a receiver operating characteristic curve (ROC) was used to determine the accuracy of the model based on true positives and negatives in total.

#### 2.2.2 Artificial neural networks

Mathematically, we can express the above using the following mathematical formula:


$$y(x)=f\left(\sum_{i=1}^{n}w_{i}X_{i}\right)$$

Theoretically, there are many types of ANNs, but each one has largely the same basic characteristics:

− Activation function: It is based on the transformation of many neurons’ input into a single output signal. This mechanism is similar to linear regression models.
− Network architecture: This describes the structure of neurons in the model, how they are connected and the number of layers. Layers are structures in which inputs and outputs are organized. A single layer denotes a simple pattern of linear type and is easily separable; on the opposite, several layers are more complex structures. There are also hidden layers that increase the complexity of ANN by allowing more connections.
− Direction: These networks also have a very specific direction. When the direction goes from input to output, then the network is called feedforward; on the contrary, the opposite is called a feedback network. Like all statistical models, also in ANNs, it is possible to calculate errors called backpropagating errors based on the backward direction which is widely used. Increasing the complexity of the model allows to increase its accuracy, as well as the relationships between input and output.

Firstly, we randomly split the data into the training and testing subgroups, and then, we scaled the data to see the overall impact on the prediction variable. We used min-max normalization that transforms the data into a common range by removing the scaling effect from all the variables. In the second step, we predicted gastrointestinal cancer using the neuronal network model. The predicted variables are scaled and need to be transformed to compare them with real values. In addition, we calculated the error in the output unit using the learning rules with the error backpropagation method. This error was backpropagated to all units so that the error in each unit was proportional to the contribution of that unit to the total error in the output unit. The errors of each unit were then used to optimize the weight of each connection. The number of hidden layers was chosen to optimize the performance (32) of non-linear transformations of the inputs entered into the network based on *N* (number of input)/2 + 3 (33). A confusion matrix was used to determine the number of true positives and negatives generated by our predictions and to summarize the performance of a classification algorithm.

The sensitivity analysis was performed to test the mean effect of the input variables on the output. After performing logistic regression and ANN, we applied the proportion test to compare the accuracy of the two methodologies. When testing the null hypothesis of no association, the probability level of error, two-tailed, was 0.05.

To increase the accuracy of ANNs and find an optimal solution for GI classification, a genetic algorithm (GA) was implemented (34). The genetic algorithm was an algorithm based on Darwin’s theory of evolution of natural selection. It was a slow gradual process that works by making changes step by step to get the best solution. Starting from a random population of ANNs with different architectures, GA changes the number of neurons in the hidden layer through the application of specific genetic operators, i.e. mutation and crossover (35).

Starting from the first generation, which could be initialized randomly or with statistical methodologies, the probability of reproduction of each individual of the population in relation to the problem was calculated using the fitness function. At this point, the crossover was carried out, i.e., the combination of the solutions for the training of the new generation. In addition to the crossover, the algorithm implemented random variations within the solutions, called mutations, in order to obtain a greater variety of individuals within the population. Optimization ended when the totality of the population converged.

To train each ANN, we balanced both the training and test sets by randomly undersampling the majority class. The first dataset was based on 150 patients negative for GI and 101 patients with GI. The second dataset contained 110 patients without GI and 101 with disease. After the choice of the number of layers with optimal number of neurons, the k-fold cross-validation was implemented, with k equal to 10, to evaluate the accuracy and the robustness of the model.

For both kinds of analysis, the GA (with and without k-fold) optimizer was used to improve the accuracy of ANN.

All statistical computations were made using StataCorp 2021 (Stata Statistical Software: Release 17; College Station, TX: StataCorp LLC) and RStudio software (“Prairie Trillium” Release).

## 3 Results

In **Table 1**, we report the patients’ baseline characteristics and nutritional intake. The mean age was 65.07 ± 8.74 and 38.25% of the patients were men. The prevalence of gastrointestinal cancers was 2.85%. Furthermore, **Table 1** shows the difference between patients with and without gastrointestinal cancer. Older patients were more prone to cancer (69.28 ± 9.56 *vs*. 64.95 ± 8.69, *p* < 0.0001) as well as those of the male gender (50.50%, *p* = 0.01). Notably, patients with a higher BMI were not more prone to cancer (25.35 ± 3.91 *vs*. 26.58 ± 4.29, *p* = 0.01). As regards nutrient intake, only in the macronutrients we found differences. Lipids and saturated and monounsaturated fatty acids were consumed less frequently by unaffected patients than by their counterparts, gastrointestinal cancer-affected patients (70.04 ± 24.44 *vs*. 76.38 ± 27.96, *p* = 0.03; 18.51 ± 8.12 *vs*. 20.41 ± 9.26, *p* = 0.02; and 36.93 ± 12.74 *vs*. 40.63 ± 15.09, *p* = 0.04, respectively).

**Table 1:** Baseline and comparison characteristics of macro- and micronutrient intake in patients with and without gastrointestinal cancer in the ONCONUT study (*n* = 3,545).

Parameters^a^	*M* ± SD or %	Gastrointestinal cancer		
		No (*n* = 3,444)	Yes (*n* = 101)	*p* ^b^
Age (years)	65.07 ± 8.74	64.95 ± 8.69	69.28 ± 9.56	<0.0001
Gender (M) (%)	1,356 (38.25)	1,305 (37.89)	51 (50.50)	0.01^c^
Educational qualification (%)				0.49^c^
Anything	1,347 (26.37)	939 (27.46)	30 (30.00)	
Primary school diploma	3,009 (58.91)	2,013 (58.88)	53 (53.00)	
Middle school diploma	492 (9.63)	317 (9.27)	11 (11.00)	
Diploma	206 (4.03)	123 (3.60)	6 (6.00)	
University degree	54 (1.06)	27 (0.79)	0 (0.00)	
Smoke (yes) (%)	381 (10.90)	372 (10.94)	9 (9.28)	0.60^c^
Marital status (%)				0.47^c^
Single	170 (4.91)	167 (4.96)	3 (3.06)	
Married or cohabiting	2,646 (76.41)	2,570 (76.37)	76 (77.55)	
Separated or divorced	32 (0.92)	30 (0.89)	2 (2.04)	
Widower	615 (17.76)	598 (17.77)	17 (17.35)	
BMI (kg/cm^2^)	26.54 ± 7.29	26.58 ± 4.29	25.35 ± 3.91	0.01
Glycemic index	56.15 ± 4.67	56.16 ± 4.66	55.57 ± 5.07	0.26
Glycemic load	135.69 ± 71.50	135.67 ± 71.01	136.39 ± 86.87	0.71
Diabetes (yes) (%)	783 (23.24)	764 (23.34)	19 (20.00)	0.45^c^
Myocardial infarction (yes) (%)	199 (6.04)	197 (6.16)	2 (2.13)	0.11^c^
Macronutrients (mg/day)^d^				
H_2_O	1,790.28 ± 730.26	1,790.53 ± 730.70	17,981.55 ± 718.72	0.96
Proteins	69.02 ± 29.40	69.11 ± 29.47	66.03 ± 27.04	0.24
Lipids	76.20 ± 27.88	76.38 ± 27.96	70.04 ± 24.44	0.03
Available carbohydrates	250.68 ± 120.90	250.62 ± 120.21	252.55 ± 143.17	0.84
Fatty acids	131.59 ± 74.07	131.57 ± 73.44	132.33 ± 93.62	0.59
Soluble carbohydrates	101.80 ± 63.81	101.77 ± 63.76	102.69 ± 65.76	0.90
Total fiber	26.26 ± 13.86	26.28 ± 13.86	25.48 ± 14.00	0.47
Saturated fatty acids	20.36 ± 9.24	20.41 ± 9.26	18.51 ± 8.12	0.02
Monounsaturated fatty acids	40.52 ± 15.04	40.63 ± 15.09	36.93 ± 12.74	0.04
Polyunsaturated fatty acids	8.30 ± 3.20	8.32 ± 3.22	7.66 ± 2.31	0.13
Cholesterol	183.64 ± 105.41	184.02 ± 105.74	170.64 ± 92.92	0.15
Alcohol	15.37 ± 19.72	15.35 ± 19.54	15.97 ± 25.02	0.59
Micronutrients (mg/day)^d^				
Na	1,447.80 ± 834.85	1,449.33 ± 825.00	1,395.78 ± 1,124.21	0.15
K	3,327.83 ± 1,654.23	3,331.27 ± 1,656.44	3,210.26 ± 1,580.59	0.54
Fe	11.18 ± 4.91	11.20 ± 4.91	10.60 ± 4.75	0.27
Ca	850.97 ± 468.05	852.18 ± 469.16	809.95 ± 428.61	0.32
P	1,143.97 ± 482.27	1,145.29 ± 482.95	1,098.83 ± 458.56	0.30
B_1_	0.78 ± 0.35	0.78 ± 0.35	0.74 ± 0.29	0.55
B_2_	1.41 ± 0.62	1.41 ± 0.62	1.33 ± 0.52	0.50
Vitamin A	1,145.28 ± 939.16	1,149.58 ± 945.67	998.74 ± 667.74	0.08
Vitamin C	170.63 ± 122.42	170.93 ± 122.60	160.30 ± 116.18	0.31

* As Mean and Standard Deviation (M±SD) for continuous variables and percentage (%) for categorical.BMI, Body Mass Index; Ψ Calculated on quantity daily consumption. § Wilcoxon rank-sum test (Mann-Whitney), ^ Chi-square or Fisher’s test, where necessary.

In **Table 2**, the association between gastrointestinal cancer and macro- and micronutrients is shown together in the model, corrected for age and gender. Only total fiber as a macronutrient resulted to have a protective role to prevent gastrointestinal cancer (OR = 0.94, 95% CI 0.89-1.00, *p* = 0.04), while K and Fe, with borderline *p*-values (OR = 1.00, 95% CI 0.99 to 1.00, *p* = 0.06, and OR = 0.87, 95% CI 0.74 to 1.02, *p* = 0.08, respectively), had a risk and a protective role, respectively, to prevent gastrointestinal cancer.

**Table 2:** Logistic regression models on the total cohort of gastrointestinal cancer (no/yes) on macro- and micronutrients, corrected for age and gender, together in the model.

	OR	se (OR)	95% CI	*p*
Macronutrients (mg/day)^a^				
H_2_O	1.00	0.0004	0.99 to 1.00	0.38
Proteins	1.01	0.01	0.98 to 1.03	0.49
Lipids	1.02	0.02	0.98 to 1.07	0.31
Available carbohydrates	1.03	0.03	0.97 to 1.09	0.37
Fatty acids	0.97	0.03	0.91 to 1.04	0.40
Soluble carbohydrates	0.98	0.03	0.92 to 1.04	0.54
Total fiber	0.94	0.02	0.89 to 1.00	0.04
Saturated fatty acids	0.94	0.04	0.87 to 1.01	0.11
Monounsaturated fatty acids	0.97	0.03	0.92 to 1.03	0.35
Polyunsaturated fatty acids	0.94	0.10	0.76 to 1.17	0.60
Cholesterol	0.99	0.002	0.99 to 1.00	0.80
Alcohol	0.99	0.01	0.98 to 1.01	0.42
Micronutrients (mg/day)^a^				
Na	1.00	0.0001	0.99 to 1.00	0.33
K	1.00	0.0002	0.99 to 1.00	0.06
Fe	0.87	0.07	0.74 to 1.02	0.08
Ca	0.99	0.001	0.99 to 1.00	0.32
P	1.00	0.001	0.99 to 1.00	0.34
B_1_	0.67	0.67	0.09 to 4.77	0.69
B_2_	0.88	0.53	0.27 to 2.88	0.84
Vitamin A	0.99	0.0002	0.99 to 1.00	0.38
Vitamin C	0.99	0.002	0.99 to 1.00	0.50

OR, odds ratio; se (OR), standard error of OR; 95% CI, confidence interval at 95%.

a Calculated on quantity daily consumption.

**Figure 2:**
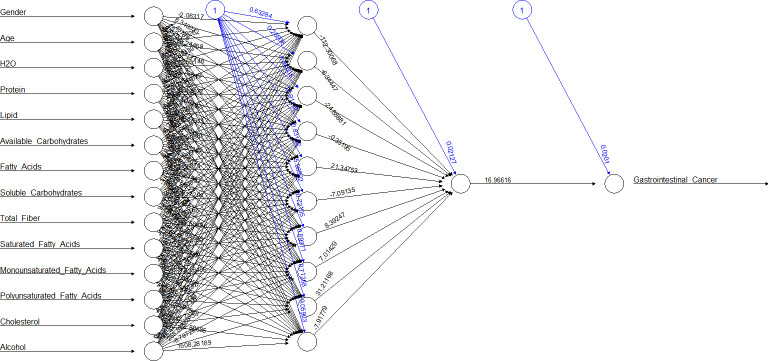
The ANN on the training dataset of macronutrients.

**Figure 3:**
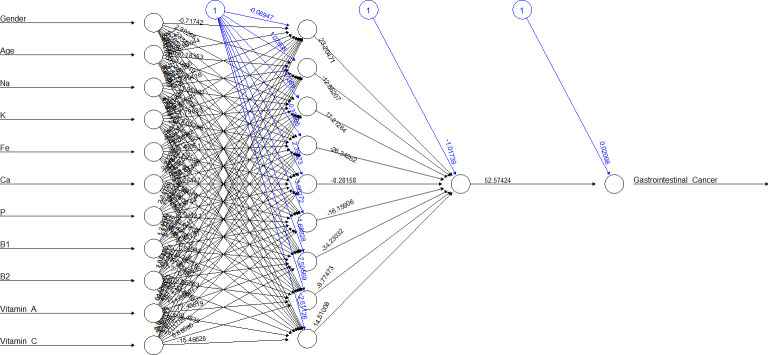
The ANN on the training dataset of micronutrients.

**Figures 2**, **3** show the ANN approach for macro- and micronutrients separately. The ANN included 10 neurons for macronutrients and 9 for micronutrients. The blue circles with arrows indicate the biases corresponding to the intercept in a typical regression model, while the black circles with arrows are the synaptic weights applied to each input variable (**Table 3**). The total error was 2.70e+01 and the steps were 1.51e+04 for macronutrients, while the error for micronutrients was 2.80e+01 and the steps were 4.59e+04.

**Table 3:** Weight values of ANN between the input and hidden layers for predicting gastrointestinal cancer on the training subset.

Inputs	Neuron 1	Neuron 2	Neuron 3	Neuron 4	Neuron 5	Neuron 6	Neuron 7	Neuron 8	Neuron 9	Neuron 10
Macronutrients										
Gender (F)	−2.06e+00	6.35e+00	2.46e+00	1.52e+01	3.12e+00	3.75e+01	3.19e+00	−1.28e−01	−7.12e+02	−7.57e+00
Age	1.89e+00	−2.45e−01	−3.63e+00	1.97e+01	1.00e+00	1.20e+01	1.64e+01	5.96e+00	−1.25e+00	−9.46e−02
H_2_O	1.82e+00	2.25e+01	−2.77e+00	2.04e+01	−1.31e+00	−1.56e+00	−2.48e+00	−8.17e+00	−5.11e+00	−5.12e+01
Proteins	2.92e+00	−2.54e+01	−1.48e+00	2.11e+01	−3.17e+00	−1.522e+00	−6.08e+00	2.60e+00	−9.70e−01	−8.17e+00
Lipids	−2.55e+00	−2.07e+01	9.24e−01	1.95e+01	−4.00e+00	−6.14e+00	−2.99e+00	−1.93e+00	5.64e+00	4.99e+00
Available carbohydrates	−1.74e+00	−8.68e−01	1.36e+00	1.89e+01	−3.83e−01	−4.51e+00	2.04e+00	4.74e−01	2.24e+00	7.15e+01
Fatty acids	−6.68e+00	2.46e+01	6.11e−01	1.91e+01	4.39e+00	−2.645e+00	−4.11e+00	−2.35e+01	5.21e+00	4.00e+01
Soluble carbohydrates	−1.75e+00	−1.56e+01	−2.69e+00	2.14e+01	3.89e+00	−1.17e+00	3.48e+00	3.77e+00	−3.82e+00	5.05e+01
Total fiber	2.20e+00	7.68e+01	−3.19e+00	2.36e+01	4.20e−02	4.98e−03	−1.56e+01	4.98e+00	−4.94e+00	9.95e+01
Saturated fatty acids	−2.13e−01	−1.33e+01	3.75e+00	2.09e+01	−1.01e+01	3.36e+00	2.25e+01	−4.50e+00	2.68e+00	6.89e+00
Monounsaturated fatty acids	−6.95e+00	−2.85e+01	1.44e+01	2.21e+01	1.32e−01	−2.76e+00	−2.62e+01	−1.82e+01	1.05e+00	−1.03e+01
Polyunsaturated fatty acids	−1.39e+01	−7.33e+01	1.30e+00	2.18e+01	−2.79e+00	−6.09e+00	−2.53e+00	8.83e+00	−2.34e+00	−8.02e+00
Cholesterol	−1.30e+01	−1.34e+01	1.81e+00	2.14e+01	−1.55e+01	7.57e+00	−9.92e+00	−1.84e+01	−2.79e+00	−2.29e+01
Alcohol	3.10e−01	2.18e+02	−4.10e+00	2.11e+01	−2.27e+01	−7.17e+00	1.54e+01	2.69e+02	−8.80e+00	1.51e+03
Micronutrients										
Gender (F)	−7.17e−01	2.99e+00	1.23e+00	4.70e−01	−2.68e−01	4.89e−01	3.35e+00	−3.21e+01	3.44e+01	
Age	−2.93e+00	−1.83e−01	3.05e+00	2.25e+00	−4.42e+00	−2.99e+00	−2.76e+00	8.58e−01	6.41e+00	
Na	−9.54e+00	−2.19e+00	1.39e+01	−1.59e+01	1.15e+00	2.48e+00	2.88e+00	4.92e+01	4.79e+01	–
K	3.96e+00	−2.67e+00	−1.80e+00	2.70e+01	−1.13e+00	−6.63e−01	−6.38e+00	−1.33e+01	−4.82e−01	–
Fe	−4.10e+00	−3.30e+01	1.38e+00	5.48e+00	1.17e+01	2.48e+00	8.40e−01	1.02e+02	−1.83e+01	–
Ca	−2.22e+00	2.38e+01	−2.26e+01	−5.86e+01	2.64e−01	7.99e+00	8.09e−02	−1.20e+01	−1.84e+01	–
P	2.32e+00	2.53e+01	−1.38e+01	−6.23e+00	6.56e+00	2.34e+00	−1.48e−01	−1.47e+01	−8.48e+00	–
B_1_	1.24e+00	1.17e+00	4.80e+00	9.38e+00	−3.60e+01	−1.82e+00	1.87e+00	−3.36e+00	−2.75e+01	–
B_2_	7.07e+00	1.33e+00	−7.76e−01	2.95e+00	−7.31e+00	−3.66e+00	−5.65e−01	−3.59e+00	−2.46e+01	–
Vitamin A	6.10e+01	−1.63e+00	1.94e+01	2.22e+01	1.52e+02	2.55e+01	1.10e+01	7.74e+01	3.05e+01	–
Vitamin C	−2.91e+00	−3.09e+00	2.31e+01	1.35e+01	1.92e+00	2.88e+00	3.02e+00	6.68e+00	−1.55e+01	–

The sensitivity analysis (**Supplementary Material 1**) that lipids, cholesterol, and saturated fatty acids (0.0059154648, 0.0052250640, and 0.0042273502, respectively) as macronutrients and B_1_, B_2_, and P (0.0064871691, 0.0061667545, and 0.0051315392, respectively) as micronutrients had the best influence in predicting outcome. **Table 4** compares the two methods in terms of accuracy rate based on the confusion matrix (true positives and true negatives in total). The accuracy for macronutrients was 97.15% with LR and 96.61% with ANN, while for micronutrients, it was 97.15% with LR and 97.06% with ANN; no statistically significant difference was elicited (*p* = 0.39 and *p* = 0.85).

**Table 4:** Accuracy table based on the confusion matrix of LR and ANN to predict gastrointestinal cancer, in macro- and micronutrients.

Parameters	Accuracy (%)		
	LR	ANN	*p*
Macronutrients	97.15	96.61	0.40
Micronutrients	97.15	97.06	0.88

Considering the balanced datasets **Table 5** reports the obtained performances. We firstly investigated the discrimination capabilities of two ANN architectures with one hidden layer with 30 neurons for macronutrients and micronutrients. In the first case, we reached accuracy of 72%, whereas in the second case we obtained an accuracy of 74%. Subsequently, the GA-based optimization allowed us to find different optimal solutions for both the classification tasks optimizing the accuracy and AUC values. **Table 5** shows the optimal ANN configurations which, for the classification of macronutrients, had two hidden layers, with 24 and 82 neurons, respectively, that allowed to obtain an accuracy of 76.20%; instead, the optimal configuration for the classification of micronutrients included two hidden layers with 99 and 121 neurons, respectively, which allowed reaching an accuracy of 73.8%. Performing cross-validation with k=10, the average accuracy had a physiological decrease, probably due to the small sample size in each test of the folds.

**Table 5:** Test results of the neural network structure optimized by genetic algorithm on balanced datasets.

							Cross-validation (k-fold = 10)	
Neurons	TP	FN	TN	FP	Accuracy (%)	AUC (%)	Accuracy (%)	AUC (%)
Macronutrients (150/101)								
30	11	9	25	5	72.00	72.50	59.00	61.00
Macronutrients (110/101)								
								
24,82^a^	17	3	15	7	76.20	73.90	57.40	56.90
Micronutrients (150/101)								
30	11	9	26	4	74.00	77.00	59.70	58.60
Micronutrients (110/101)								
99,121^a^	16	4	15	7	73.80	80.60	55.50	59.00

a Obtained with genetic algorithm.

## 4 Discussion

In this paper, comparing the accuracy of the logistic model with ANN in an elderly cohort from Southern Italy, there was no statistically significant difference between the two techniques, although in some cases in the literature, the classic logistic model resulted slightly better than ANN for both macro- and micronutrients.

For many authors, the logistic model is a particular case of ANN with one layer. It allows to determine easily the variables that were predictive of the outcome on the basis of coefficients and the corresponding odds ratios (36). It is a model where there is a direct relationship between the input variables and the probabilistic outcome, unlike ANN, where at each level a logistic model is built. ANN is a semiparametric method with many advantages, being useful to handle a large number of variables in the model, with no need to make assumptions of a normal distribution, and for the detection of a complex and non-linear relationship between independent and dependent variables (37); in fact, medical outcomes are dependent on a variety of factors such as the patient’s age, gender, smoking, or family history (38). LR is easier to implement and interpret. It makes no assumptions about distributions of classes in the feature space and provides a measure of how appropriate a predictor is and also its direction of association. LR requires average or no multicollinearity between independent variables. More powerful and compact algorithms such as ANN can easily outperform this problem.

Concerning ANN, inspired by the behaviour of a human brain, it is capable of performing more complex tasks and activities as compared to other approaches. The other advantage of ANN is that its structure is adaptive in nature, i.e. ANN architecture could be adapted depending on the classification purpose.

In the opposite, the ANN performances depend on the amount and quality of data it receives for training. Although many studies have demonstrated that ANN has a better performance than LR, ANN has some disadvantages, such as the dependency of the performances from the sample size of the training set or, the number of hidden layers, difficulties in interpretation (39), and the need of experience by the biostatistician that performs the data analysis. Improving the predictive accuracy of ML models and assessing their applicability in various clinical situations remain important challenges (40). The application of the ANN model is of great significance in public health. It could be used as a preliminary screening tool to identify individuals at high risk of cancer based on their dietary factors; it could also guide the prevention strategy in clinics (41).

In this study, the number of variables relative to the sample size was not large. The fiber intake in our study had a protective role as in several studies in the literature (42, 43), although not statistically significant. In contrast, high levels of K were associated with the risk of developing the disease (44). The abundance of fiber intake had a protective role against gastrointestinal cancer, while refined grains, rich in available carbohydrates, were associated with an increased risk of rectal cancer (45, 46). Fiber intake determines the composition and function of the gut microbiota and plays a critical role in the maintenance of colonic health through fermentation (47). In the opposite, the available carbohydrates were associated with higher glycemic load (48) that conducts to higher blood glucose and insulin responses and metabolic dysregulation (49). About the role of fatty acids and monounsaturated fatty acids (50) seems that regulate the reduction of cell proliferation and increase apoptosis, but it is not well understood now (51).

If we considered ANN represented in **Table 3**, for example, neuron 1, we can see how the high weights of fibers for macronutrients and potassium for micronutrients were in ANN, others variables appeared in relation with gastrointestinal cancer, but this is the advantage of this statistical methodic, that allows to create multiple relations between the variables involved respect to LR. In the opposite, creating an ANN was more difficult respect to setting up a logistic regression model, and the choice of best hyperparameters was very difficult.

Being able to predict the consumption of macronutrients and micronutrients, i.e., the eating habits of a cohort in a particular geographical area, is interesting because it will allow in the future to use simpler but more useful tools for personalized medicine.

However, we must consider that this study had limitations in terms of sample size and the prevalence of gastrointestinal cancer cases. We considered a very large range of ICD-10, and it cannot be excluded that in the future, stratifying by single pathology, the performance of ANN may significantly increase.

This was a preliminary study conducted in an elderly cohort in Southern Italy that started 30 years ago, which could be compared in the future with a more recent cohort from the same geographic area.

The follow-up patients included in the historical cohort could be useful for studying the change in eating habits (as well as macro- and micronutrient intake) over time. Furthermore, it could be useful to verify if the validity of these new techniques applied to an old cohort can also be found on the current population which could be useful for possible validation and replication. Moreover, the historical cohort was useful for future studies because it allows to study the appearance of new cases of the disease and is therefore useful for building more advanced predictive models.

## 5 Conclusion

The results of this study demonstrated that both the ANN and LR models performed well. It was difficult to draw conclusions about the superiority of one model over the other based on this study and other studies in the literature. Each model had advantages and disadvantages. In medical diagnosis, neither of the two mathematical models could replace the other, but the two models could be used to make decisions. The models could be useful in the future for understanding cancer risk factors, risk estimation, and future diagnosis accompanied by better performance of the statistical software and their complexity and applicability.

In our case, the tested algorithms can perform with high precision, sensitivity, and specificity despite substantial differences in how they are mathematically built. This was especially important because without a clear understanding of how algorithms were trained, doctors risk over-reliance on these tools which may not always work as intended. Furthermore, these data were also useful to demonstrate an important principle of machine learning, i.e., more complex algorithms do not always generate more accurate predictions; therefore, practical knowledge of the construction is useful, as in this case, to choose the most suitable model for our cohort under study and especially based on the outcomes of interest.

## Data availability statement

The datasets presented in this article are not readily available because the data are owned by IRCCS “S. de Bellis.” Requests to access the datasets should be directed to rossella.donghia@irccsdebellis.it.

## Ethics statement

The studies involving human participants were reviewed and approved by IRCCS “S. de Bellis.” The patients/participants provided their written informed consent to participate in this study.

## Author contributions

Conceptualization, GM and RD; methodology, RD, VG, CL, AB, and VB; software, RD,VG, CL, AB, and VB; formal analysis, RD, VG, CL, AB, and VB; investigation, GM; resources, VG; data curation, RD and VG; writing –original draft preparation, RD; writing –review and editing, RD and VG; visualization, VG; supervision, VG; project administration, GM. All authors have read and agreed to the published version of the manuscript.
