# Supplementary material for: Prediction of gastrointestinal cancers in the ONCONUT cohort study: comparison between logistic regression and artificial neural network
Source: Front Oncol. 2023 Apr 24;13:1110999. doi: 10.3389/fonc.2023.1110999 (PMC10166229; doi:10.3389/fonc.2023.1110999)
Supplement: Supplementary file 1 [file Table_1.docx]

Supplementary Material 1. Sensitivity analysis of ANN.

| Parameters | Mean Sensitivity |
| --- | --- |
| Macronutrients |  |
| Gender | 0.0135500694 |
| Age | 0.0073768459 |
| H2O | 0.0040239259 |
| Proteins | 0.0035806748 |
| Lipids | 0.0042273502 |
| Available Carbohydrates | 0.0013988592 |
| Fatty Acids | 0.0001488966 |
| Soluble Carbohydrates | 0.0015046927 |
| Total Fiber | 0.0021050663 |
| Saturated Fatty Acids | 0.0052250640 |
| Monounsaturated Fatty Acids | 0.0016719799 |
| Polyunsaturated Fatty Acids | 0.0012526755 |
| Cholesterol | 0.0059154648 |
| Alcohol | 0.0006540310 |
| Micronutrients |  |
| Gender | 0.0142625309 |
| Age | 0.0155334431 |
| Na | 0.0024150129 |
| K | 0.0049259031 |
| Fe | 0.0048941603 |
| Ca | 0.0003801916 |
| P | 0.0051315392 |
| B1 | 0.0064871691 |
| B2 | 0.0061667545 |
| Vitamin A | 0.0036431971 |
| Vitamin C | 0.0021002810 |
